# Pain is a common problem in patients with ILD

**DOI:** 10.1186/s12931-020-01564-0

**Published:** 2020-11-11

**Authors:** Qinxue Shen, Ting Guo, Min Song, Wei Guo, Yi Zhang, Wang Duan, Yating Peng, Shanshan Ni, Xiaoli Ouyang, Hong Peng

**Affiliations:** 1 Department of Respiratory and Critical Care Medicine, the Second Xiangya Hospital of Central-South University, NO.139 Renmin Middle Road, Changsha, 410011 Hunan China; 2 Research Unit of Respiratory Disease, Central-South University, Changsha, Hunan People’s Republic of China; 3 The Respiratory Disease Diagnosis and Treatment Center of Hunan Province, Changsha, Hunan People’s Republic of China

**Keywords:** Interstitial lung disease (ILD), Short form McGill pain questionnaire (SF-MPQ), Six minutes walking test (6MWT), Brief pain inventory (BPI), Modified medical research council dyspnea scale (mMRC), Short form-36 (SF-36), Hospital anxiety and depression scale (HADS), Healthy controls (HC)

## Abstract

**Background:**

As it is less known about the prevalence and characteristics of pain in the patients with interstitial lung disease (ILD), this paper aims at determining the characteristics of the pain in the patients with ILD.

**Methods:**

Subjects with ILD and health controls with the matched ages and genders completed Short Form McGill Pain Questionnaire (SF-MPQ) and part of the Brief Pain Inventory (BPI) Short Form to elicit the characteristics of the pain. The patients with ILD were also assessed through Pulmonary Function Test, Six Minutes Walking Test (6MWT), modified Medical Research Council Dyspnea Scale (mMRC) for state of the illness and measured health-related quality of life (HRQoL) by Short Form-36 (SF-36) and psychological associations by Hospital Anxiety and Depression Scale (HADS).

**Results:**

A total of 63 subjects with ILD and 63 healthy controls (HC) were recruited in our study. The prevalence of the pain was 61.9% in ILD versus 25.3% in HC (P = 0.005) and the median score of the pain rank index (PRI) in ILD was higher than that in HC (P = 0.014). Chest (46.1%) accounted for the highest of overall pain locations in subjects with ILD. Associated clinical factors for pain intensity in the patients with ILD included exposure history of risk factors of ILD, with a longer distance of 6MWD (≥ 250 m), and a higher mMRC score (2–4). The patients with ILD and pain are more likely to suffer impaired HRQoL (P = 0.0014) and psychological problems (P = 0.0017, P = 0.044).

**Conclusion:**

The pain is common in those with ILD and the pain intensity is associated with exposure history, 6MWD, and mMRC score. The patients with ILD and pain were possibly to suffer depression, anxiety, and impaired HRQoL.

## Introduction

Interstitial lung diseases (ILDs) refer to a complex and large group of diseases that are typically characterized by the basic pathological changes of diffuse lung parenchyma, alveolar inflammation, and interstitial fibrosis with substantial morbidity and mortality [1, 2], which are somewhat challenging due to their unknown causes. Idiopathic interstitial pneumonia (IIP) and connective tissue disease-associated ILD (CTD-ILD) are common subtypes of ILDs. As the progress of diseases, patients with ILD are suffering from distressing dyspnea, progressive deterioration in exercise tolerance and impaired health related quality of life (HRQoL) in many patients’ life domains [3, 4]. HRQoL concerns a person’s life satisfaction and happiness as affected by health, including physical, psychological and social functions [5]. As it is reported, the patients with ILD and impaired HRQoL also frequently experience pain [6–8].

In 2020, the current International Association for the Study of Pain (IASP) defined pain as “An unpleasant sensory and emotional experience associated with, or resembling that associated with, actual or potential tissue damages” [9]. Depressive symptoms in ILD were reported independently correlated with pain [10], and pain was also the most common symptoms in end-of-life stage of the patients with ILD [11, 12]. However, it is less known about the prevalence, intensity, and determinants of pain in ILD, and few studies described about the pain between the patients with ILD and healthy groups.

Given the limited treatment options available to the patients with ILD, it is important to accurately identify and optimally manage coexisting conditions. A better understanding of the characteristics of pain and its influences on symptoms and physical activities will help inform approaches for prognosis of ILD. The objectives of this study were to determine the characteristics of pain (prevalence, intensity, location, and pain sensory) in patients with ILD compared with healthy, age and gender-matched control participants, the patients with ILD complicated with other respiratory disease and explore the association between pain, dyspnea, physiology function, psychological symptoms and quality of life.

## Materials and methods

### Study overview

This cross-sectional study used routinely collected data from the Second Xiangya hospital of central south university, a tertiary hospital in Changsha, Hunan province of China, between January of 2019 and January of 2020. This study was approved by the Medical Ethical Committee of the Second Xiangya Hospital of Central South University(Approved Number KL-2014-S009), and all participants gave written informed consent.

### Study population

The ILD patients were sequentially selected from among those under hospitalization and treatments at the Second Xiangya Hospital. Inclusion criteria for the ILD group included (1) met with diagnosis criterion of ILD (the diagnosis of ILD were made by certified respiratory physicians with extensive experience with the management of ILD and according to standardized criteria [13, 14]; (2) was able to offer informed consent and medical chart; (3) was a local resident aged 40–85 years; (4) was able to complete the questionnaire interview. Exclusion criteria for the ILD group included: (1) combined with other respiratory disease; (2) with serious or unstable conditions and advanced illness, such as cardiovascular, neurological, musculoskeletal diseases and cancer, who needed to be treated as inpatients; (3) with understanding barrier; (4) was not interested in this study or rejected to sign informed consent; (5) with information missing. Healthy, age-matched control participants were recruited from Health Management Center of the Second Xiangya Hospital, included in the study if he or she (1) was a local resident aged 40–85 years; (2) without respiratory or musculoskeletal history and other advanced diseases according to the medical examination report, excluded in the study if he or she (1) was with cognitive impairment and mobility limitation; (2) was not interested in this study or rejected to sign the informed consent (Additional file 1).

### Procedures

All subjects attended for 1 visit, at which time the following measures were undertaken:

Information recorded in the registry included demographic details (gender, age, BMI, diagnosis, smoking history, commodities, and use of supplemental oxygen). Pain assessment by the Short Form McGill Pain Questionnaire (SF-MPQ) and part of the Brief Pain Inventory (BPI) Short Form: SF-MPQ was used to evaluate the pain intensity of subjects, scores of different parts of SF-MPQ were calculated to characterize the pain problems [15–17]. SF-MPQ is composed of 15 representative items of MPQ, 11 of which are feelings and 4 of which are emotions. For each description, the patients were asked to rank the intensity level: 0-none, 1-mild, 2-moderate, 3-discomfort, and 4-worse. Considering that we have used SF-MPQ to measure the pain intensity, we only use BPI for the subjects to locate the pain from the body chart, which was determined by standardized body regions based on 45 anatomical areas [18, 19].

The subjects with ILD also completed the following measures:

Whether there was a history of exposure to risk factors related to ILD, the pulmonary function test (PFT) to investigate the lung volume compartment and pulmonary diffusing capacity, according to standard criteria [20–22]. Six minutes walking test (6MWT) was conducted without supplemental oxygen according to ATS guidelines in a 30-m corridor at the pulmonary unit within the hospital [23], heart rate and oxygen saturation, as measured by pulse oximetry (SpO2) were recorded at the start and end of minutes of the test. Modified Medical Research Council Dyspnea Scale (MMRC) was used to rate their levels of dyspnea during daily life, a self-measuring tool that is associated with survival in people with IPF [24] was used to response to the changes with therapy in ILD [25]. Hospital Anxiety and Depression Scale (HADS) was used to measure the severity of anxiety and depression from 14 statements, with a higher score indicating greater anxiety or depression [26]. Short Form-36 (SF-36) was used to evaluate HRQoL, which was made up of eight dimensions, 36 projects and the sum of 8 dimension scores was SFTotal score, the higher the score was, the less damage the function it would be Ref. [27].

### Statistical analysis

Continuous variables were described as mean (SD) if they were normally distributed or as median (IQR) if they were not, and categorical variables were described as counts and percentages (%). Comparisons between groups (ILD and control; ILD with or without pain; key variables) for continuous measurements were analyzed using paired t-tests, independent t-test or Kruskal–Wallis H test, with categorical data analyzed using Chi Square test. The statistical analyses were conducted using SPSS (Version.25) software (Chicago, IL, USA). P < 0.05 was considered as statistically significant.

## Results

**Fig. 1:**
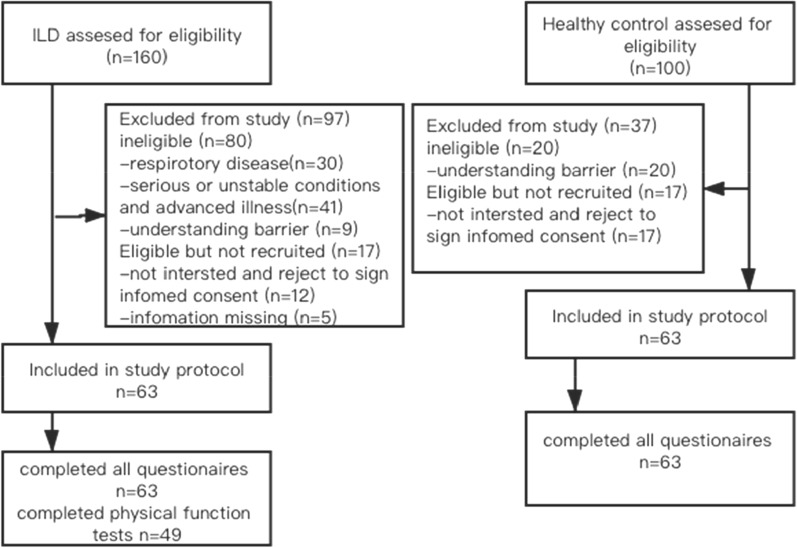
Flow diagram of study participants

From January 2019 to December 2019, a total of 141 potential subjects with ILD and 100 healthy controls were approached (Fig. 1). A total of 63 subjects with ILD and 63 healthy control subjects were recruited, with no differences in age, gender or BMI between groups. 49 patients with ILD completed physical functions and 40 patients with ILD completed 6-min walking test. The other subjects with ILD were unable to undertake the measurement due to their poor healthy situations. All subjects and healthy controls completed all questionnaires independently.

### Pain problems: ILD group versus healthy control group

The average age of all subjects was 62 ± 9 (Table1). The average pack years of the subjects with ILD was 16.68 years which was a much larger number to healthy controls whose average pack years was only 4.2 years (P < 0.0001). According to SF-MPQ, the prevalence of pain was 39 (61.9%) in ILD Group versus 16 (25.3%) in HealthControl Group (p = 0.005) and the median score of pain rank index (PRI) was 2.5 (IQR 1.0–5.0) in ILD versus 2.0 (IQR,1.0–2.0) in HC (P = 0.014). The median scores of PRI affective dimension was 2.0 (IQR 0–3.0) in ILD and 0 in HC (P < 0.0001). But the visual an analogue scales (VAS) score and present pain intensity (PPI) rank didn’t show obvious differences.

**Table 1:** Demographic and pain characteristics measured by SF-MPQ of participants

Demographic	All patients N = 126	ILD N = 63	Control N = 63	*P value*
Age (years)	62 ± 9	61 ± 10	63 ± 8	0.730
> 65	77 (61.1%)	40 (63.5%)	37 (58.7%)	0.714
Gender (Male)	77 (61.1%)	38 (60.3%)	39 (61.9%)	0.828
BMI (kg/m^2^)	24.8 ± 3.5	25.1 ± 3.6	24.4 ± 3.4	0.327
Pack years (years)	10.8 ± 17.7	16.6 ± 21.8	4.2 ± 7.1	* < 0.0001*
Pain	55 (43.6%)	39 (61.9%)	16 (25.3%)	*0.005*
Pain intensity				
PRI score	2.0 (1.0–4.0)	2.5 (1.0–5.0)	2.0 (1.0–2.0)	*0.014*
Sensory	2.0 (1.0–2.0)	2.0 (2.0–3.0)	2.0 (1.0–2.0)	0.053
Affective	1.0 (0–2.0)	2.0 (0–3.0)	0	* < 0.0001*
VAS score	4.0 (2.7–6.0)	4.5 (2.8–6.0)	4.0 (2.3–5.8)	0.163
PPI rank				
No pain(n%)	19 (34.5%)	11 (28.2%)	8 (50.0%)	0.107
Mild discomfort(n%)	29 (52.7%)	21 (53.8%)	8 (50.0%)	
Discomfort or worse(n%)	7 (12.7%)	7 (17.9%)	0	

For comparison, χ^2^ test was used for binary variables, and Student’s t-test or Wilcoxon nonparametric test was employed for continuous variables; the italic P-values indicate statistical significance

* BMI* body mass index,* PRI* pain rank index,* VAS* visual an analogue scales,* PPI* present pain intensity

**Fig. 2:**
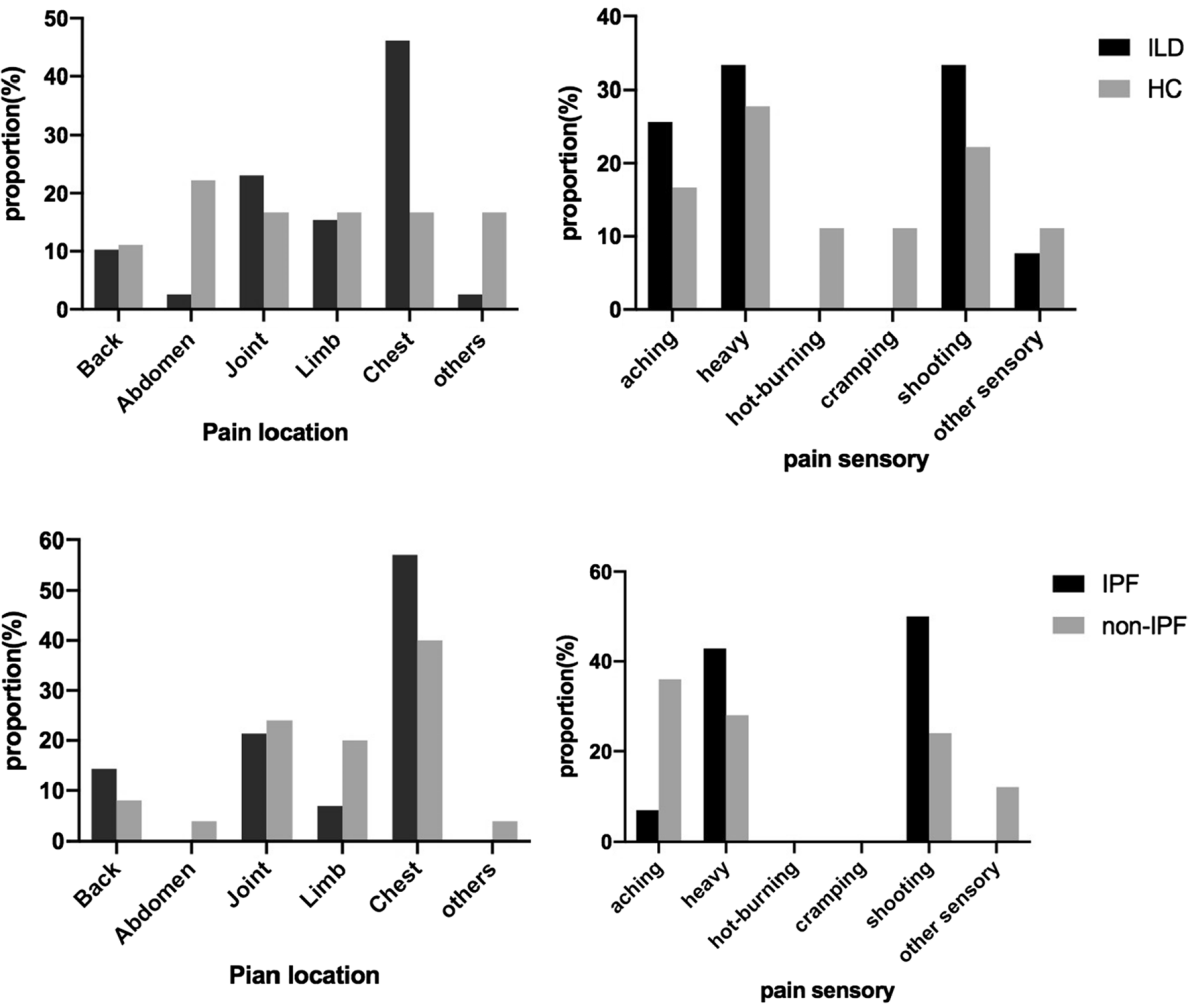
Pain location and sensory in all participants. Notes: Other pain locations include head, lumbar vertebra, Other sensory include throbbing, tender, stabbing

The proportion of pain location in subjects with HC Group and ILD Group showed significant difference in Fig. 2. In HC Group, pain locations were more evenly distributed; however, chest (46.1%) accounted for the highest of overall pain duration in subjects with ILD, and the other locations were joint (23.1%), limb (15.3%), back (10.3%), abdomen (2.6%), and head and lumbar vertebra (2.6%). Most subjects with pain problems experienced aching (25.6% in ILD Group, 16.7% in HC Group), heavy (33.3% in ILD Group, 27.8% in HC Group), shooting (33.3% in ILD Group, 22.2% in HC Group) in pain sensory. In HC Group, the subjects with pain also suffered hot-burning (11.1%) and cramping (11.1%).

### Clinical characteristics and symptom burden: Pain Versus No Pain in ILD Only

In the ILD Group (Table2), 39 (61.9%) subjects were elder than 60 years, 38 (60.3%) were male, and the average BMI was 25.1 ± 3.5 kg /m^2^, without differences between the pain and the no-pain groups. Among the 39 (61.9%) patients with ILD and pain, 14 (35.9%) was diagnosed as IPF, 22 (56.4%) was CTD-ILD and 3 (7.7%) were other ILD (two patients with NSIP and one patient with IIP). The pain locations in 14 IPF patients distributed in chest (57.1%), joint (21.4%), back (14.3%) and limb (7.1%) and in 25 non-IPF patients distributed in chest (40.0%), joint (24.0%), limb (20.0%), back (8.0%), abdomen (4.0%) and the other parts (4.0%), including head and lumbar vertebra (Fig. 2). The patients with ILD and pain had more comorbidities than the patients with ILD but without pain (P = 0.040). More patients with ILD and pain had DLCo % of predicted < 45% than the patients with ILD but without pain (P = 0.022). There was no obvious difference in FVC % of predicted, FEV1/FVC % and 6MWT in the patients with ILD with or without pain. When it came to dyspnea, the mMRC score of 32 (82.1%) patients with ILD and pain was 2–4, higher than 13 (54.2%) patients with ILD but without pain (*p* = 0.023). Besides, the subjects with ILD and pain showed more anxious and depression according to the HADS, in anxious dimension 23 (59.0%) patients with pain and 21 (87.5%) patients without pain were assessed for no case, 16 (41.0%) patients with pain and 3 (12.5%) patients without pain were assessed for borderline or case (P = 0.017); in depression dimension 23 (59.0%) with pain vs 20 (83.3%) without pain were assessed for no case, 16 (41.0%) with pain vs 4 (16.7%) without pain were assessed for borderline or case (P = 0.044). From the different dimensions of HRQL measured by RAND-36, HRQL were significantly impaired in patients with ILD and pain which can be found in SF Total score (93.0 ± 20.0 in ILD with pain, 107.5 ± 24.6 in ILD without pain, P = 0.014), and also performed in mental health (*p* = 0.029), bodily pain (P = 0.049), vitality (P = 0.043), and role emotional dimension (P = 0.006).

**Table 2:** Clinical characteristics and symptom burden of ILD patients with pain and no pain

	All patients N = 63	Pain N = 39	No-pain N = 24	*P value*
Age	61 ± 10	62 ± 10	61 ± 11	0.437
> 60 years	39 (61.9%)	23 (59.0%)	16 (66.7%)	0.601
Gender (Male)	38 (60.3%)	24 (63.2%)	14 (58.3%)	0.801
BMI (kg/m^2^)	25.1 ± 3.5	25.4 ± 3.8	24.6 ± 2.8	0.093
Diagnosis				
IPF	24 (38.1%)	14 (35.9%)	10 (41.7%)	0.882
CTD-ILD	34 (54.0%)	22 (56.4%)	12 (50.0%)	
Others	5 (7.9%)	3 (7.7%)	2 (8.3%)	
Smoker	28 (44.4%)	19 (48.7%)	9 (37.5%)	0.441
Exposure history	23 (36.5%)	17 (43.6%)	6 (25.0%)	0.181
Comorbidity	34 (53.9%)	25 (64.1%)	9 (37.5%)	*0.040*
FVC, % predicted	74.5 ± 21.8	70.8 ± 15.8	80.3 ± 28.3	0.195
< 80	32 (65.3%)	22 (73.3%)	10 (52.6%)	0.138
≥ 80	17 (34.7%)	8 (26.7%)	9 (47.4%)	
DLCO, % predicted	45.2 ± 14.2	44.1 ± 12.8	46.8 ± 16.5	0.525
< 45	23 (48.9%)	18 (62.1%)	5 (27.8%)	*0.022*
≥ 45	24 (51.1%)	11 (37.9%)	13 (72.2%)	
FEV1/FVC, %	74.7 ± 26.1	74.4 ± 25.3	75.1 ± 28.0	0.572
< 70	4 (8.1%)	4 (10.3%)	0	0.105
≥ 70	45 (91.9)	35 (89.7%)	10 (100.0%)	
6MWT	333.2 ± 112.2	329.7 ± 100.7	338.5 ± 129.0	0.801
6MWD < 250 m	9 (14.2%)	5 (55.6%)	4 (44.4%)	0.893
6MWD ≥ 250 m	31 (49.2%)	18 (58.1%)	13 (41.9%)	
6MWT SpO2, before (%)	94.5 ± 3.8	94.7 ± 3.4	94.1 ± 4.4	0.654
6MWT SpO2, later (%)	88.7 ± 8.4	89.9 ± 8.5	88.2 ± 8.5	0.809
mMRC score				
0–1 (n%)	24 (38.1%)	7 (17.9%)	11 (45.8%)	*0.023*
2–4 (n%)	39 (61.9%)	32 (82.1%)	13 (54.2%)	
HAD anxious				
No case (n%)	43 (68.2%)	23 (59.0%)	21 (87.5%)	*0.017*
Borderline and case (n%)	19 (30.2%)	16 (41.0%)	3 (12.5%)	
HAD depression				
No case (%)	43 (68.2%)	23 (59.0%)	20 (83.3%)	*0.044*
Borderline and case (%)	20 (31.7%)	16 (41.0%)	4 (16.7%)	
SF-36				
Social functioning	7.7 ± 2.5	7.3 ± 2.4	8.1 ± 2.4	0.265
Mental health	20.1 ± 5.8	18.9 ± 5.3	33.0 ± 6.1	*0.029*
Bodily pain	10.4 ± 4.3	9.5 ± 4.1	11.7 ± 4.2	*0.049*
Vitality	14.4 ± 5.6	13.2 ± 5.6	16.2 ± 5.5	*0.043*
Role emotional	3.8 ± 1.3	3.4 ± 1.1	4.3 ± 1.3	*0.006*
Physical functioning	17.1 ± 8.4	16.2 ± 8.1	18.6 ± 9.1	0.294
General health	16.3 ± 3.6	16.1 ± 3.5	16.8 ± 3.8	0.474
Role physical	6.2 ± 4.0	5.7 ± 3.7	7.0 ± 4.4	0.218
Health Transition	2.5 ± 1.1	2.4 ± 1.1	2.5 ± 1.2	0.661
SF Total score	98.7 ± 22.9	93.0 ± 20.0	107.5 ± 24.6	*0.014*

For comparison, χ^2^ test was used for binary variables, and Student’s t-test or Wilcoxon nonparametric test was employed for continuous variables; the italic P-values indicate statistical significance

* 6MWT* Six minutes walking test,* MMRC* Modified Medical Research Council Dyspnea Scale,* HADS* Hospital Anxiety and Depression Scale,* SF-36* Short Form-36

### Pain characteristics and related factors according to SF-MPQ

Table 3 presents pain characteristics from SF-MPQ according to key variables. There is no obvious difference of the pain intensity in the patients with ILD with age (divided line as 60 years old) and gender. Patients with a history of exposure to risk factors related to ILD had a significantly higher score in the sensory dimension (P = 0.010). Patients with higher mMRC score (ranging 2–4) showed greater pain intensity indicated by sensory dimension (P = 0.014), affective dimension (P = 0.037), and PRI (P = 0.006). Patients whose 6MWD ≥ 250 m had a significantly higher score of pain in PPI rank than the patients who had 6MWD < 250 m (P = 0.005). The pain intensity of the patients with ILD showed no difference in FVC% predicted, DLCO% predicted and FEV1/FVC%. The patients with ILD assessed by HADS on no case showed lower pain intensity than the patients assessed on borderline or case both in anxious dimension indicated by sensory dimension (P = 0.002), affective dimension (P = 0.001), PRI (P = 0.001), VAS(P = 0.017) or PPI (P = 0.014) and in depression dimension indicated by sensory dimension (P = 0.002), affective dimension (P = 0.001), PRI (P < 0.0001), VAS (P = 0.013) or PPI (P = 0.046).

**Table 3:** Pain characteristics from SF-MPQ according to key variables

	PRI score			VAS	PPI rank (N%)		
	Sensory	Affective	Total	Score	No pain	Mild discomfort	Discomfort or worse
Age							
< 60 years	1.5 (0–3.0)	0 (0–2.0)	2.5 (0–5.0)	3.0 (0–5.7)	2 (12.5)	9 (56.3)	5 (31.3)
≥ 60 years	1.0 (0–2.0)	0 (0–2.0)	2.0 (0–4.0)	3.0 (0–50)	3 (13.0)	13 (56.5)	7 (30.4)
* P-value*	0.308	0.828	0.607	0.456	0.998		
Gender							
Male	2.0 (0–3.0)	0 (0–2.25)	2.0 (0–5.0)	3.0 (0–6.0)	4 (16.7)	10 (41.7)	10 (41.7)
Female	1.0 (0–2.0)	0 (0–2.0)	2.0 (0–4.0)	3.0 (0–4.5)	1 (6.7)	12 (80.0)	2 (13.3)
* P-value*	0.344	0.969	0.541	0.906	0.063		
Exposure history							
No	0.5 (0–2.0)	0 (0–2.0)	1.0 (0–4.0)	3.0 (0–3.8)	3 (13.6)	13 (59.1)	6 (27.3)
Yes	2.0 (0–3.0)	1.0 (0–4.0)	3.0 (0–6.0)	5.0 (0–6.0)	2 (11.8)	9 (52.9)	6 (35.3)
* P-value*	*0.01*	0.057	0.505	0.143	0.865		
6MWD							
< 250 m	0.5 (0–2.0)	0 (0–2.0)	1.0 (0–4.0)	3.0 (0–3.8)	2 (40.0)	1 (20.0)	2 (40.0)
≥ 250 m	2.0 (0–3.0)	1.0 (0–4.0)	3.0 (0–6.0)	5.0 (0–6.0)	0 (11.1)	15 (88.9)	3 (16.7)
* P-value*	0.849	0.633	0.406	0.824	*0.005*		
mMRC score							
0–1	0 (0–1.0)	0 (0–0.5)	0 (0–1.5)	0 (0–3.4)	1 (14.3)	3 (42.9)	3 (42.9)
02-Apr	2.0 (0–2.0)	1.0 (0–2.0)	3.0 (1.0–5.0)	3.0 (0–5.3)	4 (12.5)	19 (59.4)	9 (28.1)
* P-value*	*0.014*	*0.037*	*0.006*	0.104	0.706		
FVC, % predicted							
< 80	1.0 (0–2.0)	0 (0–2.0)	2.0 (0–5.0)	3.0 (0–5.0)	4 (18.2)	13 (59.1)	5 (22.7)
≥ 80	1.5 (0–2.0)	0 (0–2.0)	2.0 (0–4.3)	3.0 (0–5.3)	1 (12.5)	6 (75.0)	1 (12.5)
* P-value*	0.728	0.908	0.955	0.605	0.721		
DLCO, % predicted							
< 45	2.0 (0–2.5)	0 (0–2.5)	3.0 (1.0–5.0)	3.0 (0–5.3)	2 (11.1)	12 (66.7)	4 (22.2)
≥ 45	1.0 (0–2.0)	0 (0–2.0)	1.0 (0–4.0)	3.0 (0–5.0)	3 (27.3)	6 (54.5)	2 (18.2)
* P-value*	0.231	0.517	0.099	0.333	0.535		
FEV1/FVC, %							
< 70	3.0 (2.0–4.0)	3.0 (2.0–4.0)	5.0 (4.0–8.0)	6.0 (3.0–7.0)	5 (19.2)	17 (65.4)	4 (15.4)
≥ 70	2.5 (2.0–3.0)	1.5 (0–3.7)	4.0 (3.0–5.7)	6.0 (3.5–7.0)	0	2 (50.0)	2 (50.0)
* P-value*	0.225	0.169	0.999	0.598	0.226		

For comparison, χ^2^ test was used for binary variables (*Fisher’s test) and Student’s t-test or Wilcoxon nonparametric test was employed for comparisons of two independent groups of continuous variables

## Discussion

Pain problems were prevalent in the patients with ILD but only few studies were performed for this problem. It is the first time that study was performed to measure pain problems in the patients with ILD by SF-MPQ and a healthy control group, persons of similar age and gender without lung disease, were set in order to explore the characteristics of pain in the patients with ILD. In our present study, these findings indicate that pain was found commonly in both IPF and non-IPF ILDs, and the prevalence of this deficit was higher compared with the rates found in healthy controls. The main pain location in the patients with ILD was chest, joint and limb. The intensity of pain may be related to exposure history, 6MWD and mMRC score. Compared with the patients with ILD but without pain, patients with pain also experienced impaired physical and mental health status, which might be predominantly caused by more limitations in daily functions.

Significant pain is not considered as a typical characteristic of ILD. However, in some large cohort studies, higher prevalence of pain was found in ILD compared with that in the general population [28, 29]. It is in line with our findings that more than half patients with ILD suffered pain in their daily life, which is much higher than that in healthy controls. When it came to the other chronic diseases, pain is also mentioned frequently but most chronic diseases show more prevalent pain problems. A meta-analysis, including 1,571 articles were identified, reporting that pain prevalence of COPD in high-quality studies ranged from 32 to 60%, and comorbidity, nutritional status, QoL and several symptoms were related to pain [30]. The prevalence of pain in patients with advanced CKD had been estimated at approximately 50%–70% [31]. In 2018, a large healthy study in Norway [32], with 50,807 subjects, found that 43.0% in the diabetes group, and 75.4% in the arthritis group suffered from chronic pain, and a large European survey also reported arthritis was the most common cause of pain, followed by COPD and heart disease [29]. More control groups with chronic diseases should be added in the future study to compared with the specifics of pain in ILD.

In the ILD individuals, the main pain locations were chest (46.2%) and joint (23.1%) among all the patients with ILD and pain. 57.1% of the patients with IPF and pain declared having chest pain, which was higher than non-IPF (40.0%). Kaisa’s study [8] also found that 31.2% (79/253) patients with IPF experienced chest pain. But a British study [33] about 111 patients with fibrotic ILD found that most frequently reported painful areas of these subjects were the back (34%) and lower limbs (25%), and they were similar compared with the patients with and without IPF. According to literatures about pulmonary disease, the causes of chest pain remain unclear which can be related to pulmonary loss of elasticity of the parietal pleura, pathological bronchial fibrosis, thoracic vertebral deformity, costotransverse, intervertebral arthropathy and activities related to breathing and postural dysfunction [34, 35]. The incidence of joint pain in patients with CTD-ILD was higher than in the patients with IPF in our study, which was in line with the previous studies [36]. It was reported that the prevalence of joint pain in CTD-ILD patients could be explained by the high anti-cyclic citrullinated peptide antibody (anti-CCP) positivity in patients [37].

ILD Group experienced a higher pain intensity than HC Group both in feeling and emotion dimension. A higher intensity of pain in the patients with ILD was also associated with many factors in our study, including exposure history of ILD risk factors, longer distance of 6MWD (≥ 250 m), higher mMRC score (2–4) and impaired SF-36 and HAD score. When undergoing severe dyspnea, the patients normally gave extra worse results of pulmonary function test, especially FVC, % predicted and DLCo, % predicted, and unsatisfied 6WMT, a practical and reliable measure of exercise tolerance that was widely used to assess the functional status of the patients with IPF [38], which showed the severity of the patients’ current conditions and reflect the current quality of life [39–41]. It was reported in previous studies that the association between dyspnea severity in mMRC score and intensity of pain was reported in the previous studies [8, 11], and the prevalence of chest pain in the patients with IPF had a positive linear relationship to increased mMRC score [8]. In our study, compared with the patients with ILD but without pain, the ILDs with pain did have a higher mMRC score. Moreover, according to the results of MPQ, the pain intensity in the patients with ILD was greatly infected by dyspnea severity. But we didn’t see the relationship of pain intensity in the patients with ILD with the 6MWT SpO2 and FVC, % predicted, and DLCO, % predicted. Given that the patients with COPD showed obvious chronic pain and some patients with ILD also showed FEV1/FVC, % decline under 70% in the as the disease progress, we compared the intensity of pain in the patients with ILD, who were confirmed to experience pain problems but there were only negative results. Even the severity of dyspnea may have impacts on the intensity of pain [42], no apparent paradoxical relation between pain intensity of COPD and lung function (FEV1 and FEV1 percentage predicted) had been reported in previous pain studies on COPD [30, 43–45]. This inverse relationship, was also probably caused by selection bias, also could be interpreted that other symptoms like dyspnea were more distressing than pain, leading to more focuses on dyspnea and less on pain, also causing patients to be reluctant to spontaneously report pain [46–48].

The patients with ILD and pain suffered worse quality of life and psychological deficits, like symptoms of anxiety and depression [44, 45]. The impaired HRQoL, according to results of SF-36, except for the poor total score, was mainly performed on mental health, bodily pain, vitality, and role emotional, which was reflected in the results of SF-MPQ and HADs. We further found that the pain intensity was related to the degree of depression and anxiety. In addition to increasing dyspnea, many of the ILDs, such as sarcoidosis and connective tissue disease ILDs, are associated with extrapulmonary manifestations that may also lead to pain and add tremendous burden on HRQoL and mental health. [49, 50] Ryerson et. al. [10] reported the novel findings that baseline pain severity was associated with baseline depression score and particularly in the non-idiopathic pulmonary fibrosis population*.* Therefore, those indicated the need of healthcare providers, clinicians, and patients to pay greater attention to the patients with ILD and pain and consider strategies to minimize their impacts on the patients’ quality of life, healthcare utilization, and prognosis.

﻿To our knowledge, this is the first study to investigate pain in patients with ILD, including the intensity, location, type and associated factors. However, generalizability beyond this specific group and setting is limited, as only 126 subjects from one hospital were included, there are some limits in our study results to a single time-point and does not allow us to describe the changes in pain or symptoms over time. Our study may be subjected to some selection bias and as some patients at a very advanced stage of the disease or close to death were likely to be lost from the cohort. Possibilities of false negative due to small sample size and false positive due to multiple testing. Another limitation is that the score of this questionnaire may be mixed with subjective feeling, especially the VAS score, and effected by individual verbal comprehension. The last but not the least, what was the accurate cause of pain in the patient with ILD couldn’t be completely sure in our study. In the future, a larger sample of cross- sectional or cohort studies may be conducted on factors related to pain intensity to further verify these results.

## Conclusion

In conclusion, pain is common in the patients with ILD and the pain intensity is associated with exposure history, 6MWD, and mMRC score. The patients with ILD and pain have more possibility to suffer depression, anxiety and impaired HRQoL. Knowledge of pain in ILD and intervention measures should be developed for both patients and clinicians about the pain management to improve the health-related quality of life at early stages of ILD.

## Supplementary information

**Additional file 1: Table S1.**Exclusion criteria of participants.

## Data Availability

The datasets used and/or analysed during the current study are available from the corresponding author on reasonable request.

## Abbreviations

ILD: Interstitial lung disease
IPF: Idiopathic pulmonary fibrosis
HRQoL: Health related quality of life
SF-MPQ: McGill Pain Questionnaire
BPI: The Brief Pain Inventory short form
PFT: The pulmonary function test
6MWT: Six minutes walking test
SpO2: Pulse oximetry
MMRC: Modified medical research council dyspnea scale
HADS: Hospital anxiety and depression scale
SF-36: Short form-36
PRI: Pain rank index
VSA: Visual analogue scales
PPI: Present pain intensity
