# Supplementary material for: Pain is a common problem in patients with ILD
Source: Respir Res. 2020 Nov 11;21:297. doi: 10.1186/s12931-020-01564-0 (PMC7659082; doi:10.1186/s12931-020-01564-0)
Supplement: Supplementary file 1 — Additional file 1: Table S1.Exclusion criteria of participants. [file 12931_2020_1564_MOESM1_ESM.docx]

Additional Table. Exclusion criteria of participants

| Groups | Exclusion criteria | Number |
| --- | --- | --- |
| ILD group |  |  |
|  | - combined with respiratory disease | 30 |
|  | - with serious or unstable conditions and advanced illness, such as cardiovascular, neurological, musculoskeletal diseases and cancer, who needed to be treated as inpatients | 41 |
|  | - with understanding barrier | 9 |
|  | - was not interested in this study or rejected to sign informed consent | 12 |
|  | - with information missing | 5 |
| Healthy control |  |  |
|  | - with cognitive impairment and mobility limitation | 20 |
|  | - was not interested in this study or rejected to sign informed consent | 17 |
